# Distribution of human papillomavirus genotypes in women with high-grade cervical intraepithelial lesions and cervical carcinoma and analysis of human papillomavirus-16 genomic variants

**DOI:** 10.3325/cmj.2021.62.68

**Published:** 2021-02

**Authors:** Magdalena Karadža, Snježana Židovec Lepej, Ana Planinić, Ivana Grgić, Ante Ćorušić, Pavao Planinić, Mario Ćorić, Lea Hošnjak, Kristina Fujs Komloš, Mario Poljak, Adriana Vince

**Affiliations:** 1 Department of Gynecologic Oncology, Clinic for Gynecology and Obstetrics, Clinical Hospital Centre Zagreb, Zagreb, Croatia; 2 Dr. Fran Mihaljević University Hospital for Infectious Diseases, Zagreb, Croatia; 3 Institute of Immunology and Microbiology, Faculty of Medicine, University of Ljubljana, Ljubljana, Slovenia; 4 University of Zagreb School of Medicine, Zagreb, Croatia; Karadža et al: HPV genotypes in women with high-grade cervical intraepithelial lesions and cervical carcinoma

## Abstract

**Aim:**

To analyze the distribution of high-risk human papillomavirus (HR-HPV) genotypes and the diversity of HPV-16 genomic variants in Croatian women with high-grade squamous intraepithelial lesions (HSIL) and cervical carcinoma.

**Methods:**

Tissue biopsy specimens were obtained from 324 women with histopathologically confirmed HSIL or cervical carcinoma, 5 women with low-grade SIL, and 49 women with negative histopathology. HR-HPV DNA was detected with Ampliquality HPV-type nucleic-acid hybridization assay, which identifies 29 different HPV genotypes. HPV-16 genomic variants were analyzed by an in-house sequencing.

**Results:**

The most common HPV type in women with HSIL was HPV-16, detected in 127/219 (57.9%) specimens. HPV-16 was also the dominant type in squamous cell cervical carcinoma (46/69 or 66.7%) and in adenocarcinoma (18/36 or 50.0%). Out of 378 patients, 360 had HR-HPV (282 single infections and 79 multiple infections), 3 (0.8%) patients had low-risk HPV, and 15 (4%) tested negative. HPV-16 variants were determined in 130 HPV-16 positive specimens, including 74 HSIL and 46 carcinoma specimens. In HSIL specimens, 41 distinct variants were found, 98.6% belonging to the European branch and 1.4% belonging to the African branch. In cervical carcinoma specimens, 95% isolates grouped in 41 variants belonging to the European branch, one isolate (2.5%) belonged to the North American, and one (2.5%) to the Asian-American branch.

**Conclusion:**

HPV-16, mainly belonging to the European branch, was the most frequent HPV genotype in women from Croatia with histologically confirmed HSIL and cervical cancer.

Cervical cancer is the second leading cause of death in women in low-income countries (1). Persistent infection with particular human papillomavirus (HPV) genotypes is a necessary but not a sufficient requirement for the development of cervical cancer (2). HPV DNA is detected worldwide in nearly all specimens of invasive cervical cancer, including squamous cell carcinomas, adenocarcinomas, and the majority (>95%) of immediate cervical cancer precursors (3). An epidemiological study by Bosch et al (4) has shown that the most common HPV genotypes in HSIL and squamous cell carcinomas were HPV-16, HPV-18, HPV-31, HPV-33, HPV-35, HPV-45, HPV-52, and HPV-58, with a combined worldwide relative contribution of 91% and the predominant role of HPV-16, HPV-18, and HPV-45 in cervical adenocarcinoma.

HPV genomic variants are defined as the viruses that vary by 2% or less in specified regions of the genome, and some display different oncogenicity (5). HPV-16 heterogeneity has been extensively investigated (6-12), and HPV-16 genomic variants have been identified to belong to five main branches: European, Asian-American, two African branches, and an Asian branch (13). Two subsequent studies expanded these classifications and reported a new branch: North American 1 (14,15).

Epidemiological studies have shown that non-European HPV-16 variants may promote viral persistence and disease progression (16-19). HPV-16 E6 variants, including the European HPV-16 T350G variant in the E6 gene, were detected up to 20 times more often in patients with high-grade cervical disease compared with controls. A novel HPV-16 variant, identified in Croatia, harboring a 63-bp in-frame duplication in the E1 gene, was presumed to be of reduced oncogenicity (11).

According to the several national or regional studies in women with normal and abnormal cytology, HPV-16 is the most common high-risk genotype in Croatian women (20-27). However, none of these studies involved HPV genotyping in tissue specimens, and the majority were performed in general population with a small number of women with histologically confirmed HSIL or cervical cancer. The genomic diversity of high-risk HPV genotypes in Croatia has not been studied to date. On the other hand, recommended, non-mandatory, free-of-charge, nine-valent HPV vaccine is available in Croatia and is intended for vaccination of both women and men aged 14 to 25 years (28).

The aims of this study were to analyze the distribution of high-risk HPV genotypes (HR-HPV) in women with histologically confirmed HSIL and cervical carcinoma and to analyze the genomic diversity of HPV 16 in HSIL in comparison with invasive cervical cancer.

## PATIENTS AND METHODS

### Patients and samples

The study enrolled 406 women aged 19-83 years (median 37, standard deviation [SD] 11.31) treated at the Department of Obstetrics and Gynecology, University Hospital Centre Zagreb for abnormal cytology (HSIL or carcinoma) between December 2009 and December 2013. Two tissue specimens (one for histopathological analysis and one for HPV genotyping) were obtained during colposcopy by biopsy of suspected lesions (90 specimens) or during a surgical intervention: large loop excision of the transformation zone, conization, and radical hysterectomy (316 specimens). The procedures were performed by four experienced gynecologists. The specimens were stored in the media provided within the Swab Specimen Collection Kit (Qiagen, Hilden, Germany). The histopathological evaluation was performed by two certified pathologists, who were unaware of the patients' HPV status. The histopathological tissue preparation was performed according to the standard protocol: the tissue was paraffin-embedded, sliced to 3-5 microns, and stained with hematoxylin and eosin.

The selected demographic and clinical data (age, place of residence, cytology results, risk factors for cervical cancer including smoking, parity, age of menarche) were collected with the standardized questionnaire.

The study was approved by the Ethics Committees of Department of Gynecology and the University Hospital of Infectious Diseases in Zagreb. The study was conducted according to principles of the Declaration of Helsinki. All patients signed the informed consent before entering the study.

### HPV genotyping

DNA was isolated by using DNA I Blood Cell High Performance II kit on a Magna Pure LC 2.0 instrument (Roche, Pleasanton, CA, USA) and stored at -20 °C until amplification. HPV was detected and genotyped with Ampliquality HPV-type nucleic-acid hybridization assay (AB Analitica, Padua, Italy), which identifies 29 HPV genotypes. The assay is based on the amplification of a target sequence of 150 bp within the L1 genomic region by using GP5+/6+ universal primer pair.

### Analysis of HPV-16 genomic variants

The target sequence of the L1-LCR-E6 region genome of about 1700 bp HPV-16 was amplified by using g16f-7122, g16f-7122, g16r-7714, g16f-7663, g16r-376, g16f-273, and g16r-913 primers on GenAmp PCR System 9700 (Applied Biosystems, Foster City, CA, USA) (29,30), and sequenced by using 3500 Dx Genetic Analyzer (Applied Biosystems). The presence of the amplicon was analyzed by gel electrophoresis (0.8% agarose). Nucleotide sequences were analyzed with the Vector NTI program (Thermo Fisher Scientific, Waltham, MA, USA). Nucleotide HPV-16 sequences (E6, E7, and long control region [LCR]) from patients and reference nucleotide sequences were aligned by using MAFFT, v. 6.846 software algorithm (31). A maximum likelihood phylogenetic tree was constructed with the RAxML HPC2, v. 7.6.3 algorithm (32), based on the evolutionary GTRCAT model and with the bootstrap value of 1000.

### Statistical analysis

The normality of distribution was tested with the Smirnov-Kolmogorov's test. The differences in age, age of menarche, number of births and abortions, and smoking were compared using the ANOVA test. The Pearson test was used to assess the differences in the distribution of single and multiple infections in the samples according to different pathohistological diagnoses. *P* values lower than 0.05 were considered significant. The analysis was conducted with IBM SPSS, version 25.0 (Armonk, New York, NY, USA).

## RESULTS

### Histopathologic findings

Seventy patients had a negative result, 5 had cervical intraepithelial neoplasia (CIN) 1 (mild cervical intraepithelial neoplasia – changes limited to the lower third of the epithelium), 15 had CIN 2 (moderate cervical intraepithelial neoplasia – changes involving the lower two-thirds of the epithelium), 206 had CIN 3 (severe cervical intraepithelial neoplasia – changes involving more than two-thirds of the epithelium), 24 had microinvasive carcinoma (MIC), 45 had squamous cell carcinoma, 8 had adenocarcinoma *in situ* (AIS), 20 had adenocarcinoma, 1 had adenosquamous carcinoma *in situ*, and 2 had adenosquamous carcinoma. Five patients had cancer of other origin. One specimen was a mixture of adenocarcinoma and MIC (0.3%) and 4 specimens were a mixture of CIN3 and AIS (1.1%). These specimens were classified according to the more severe clinical diagnosis as invasive lesions. The specimens of cancer of other origin were excluded from the HPV analysis, thus 401 specimens were further tested for the presence of HPV DNA.

### Human papillomavirus detection and genotyping

HPV DNA could not be detected in 6 specimens due to the poor quality of the biopsy tissue (negative internal control). In 13 specimens, HPV was not detected (histopathology of those samples was also negative). In 1 specimen with negative histopathology, we detected only low-risk HPV genotype, and in 3 specimens we detected HR HPV of unknown type. Consequently, these 23 specimens were not further analyzed.

HPV genotyping for 324 patients with histologically confirmed HSIL or cervical carcinoma, 5 patients with LSIL, and 49 patients with negative histopathological finding is shown in Table 1.

**Table 1:** Distribution of human papillomavirus (HPV) types in patients depending on histopathological finding and the number of infections

	Number (%) of samples; number of monoinfections + number of multiple infections			
HPV type	negative histopathology (n = 49)	high-grade squamous intraepithelial lesions (n = 219)	squamous cell carcinoma (n = 69)	adenocarcinoma (n = 36)
HPV-16	21 (42.9); 16 + 5	127 (57.9); 99 + 28	46 (66.7); 37 + 9	18 (50.0); 14 + 4
HPV-31	6 (12.2); 4 + 2	28 (12.8); 21 + 7	10 (14.5); 5 + 5	
HPV-51	5 (10.2); 2 + 3			
HPV-58	4 (8.2); 3 + 1	14 (6.4); 11 + 3		
HPV-33		15 (6.8); 9 + 6	4 (5.8); 2 + 2	
HPV-52		13 (5.9); 7 + 6		
HPV-18		10 (4.6); 3 + 7	5 (7.2); 3 + 2	15 (41.7); 10 + 5
HPV-45		7 (3.2); 2 + 5	5 (7.2); 4 + 1	2 (5.6); 1 + 1
HPV-low risk	2 (4)	1 (0.5)		
HPV DNA negative		8 (3.7)	3 (4.3)	4 (11.1)
Multiple infections	12 (25.5)	43 (19.6)	14 (20.3)	7 (19.4)

High-risk HPV types were detected in 360 out of 378 specimens (95.5%). Three (0.8%) specimens had low-risk HPV infection. The histopathology of HR-HPV-positive specimens was as follows: 47 specimens with negative histopathology, 5 LSIL lesions (CIN 1), 210 HSIL lesions (15 CIN 2+, 195 CIN 3), and 98 cervical carcinoma specimens (66 squamous cell subgroup +32 adenocarcinoma subgroup).

A total of 282 out of 360 (78.3%) specimens had a single infection with HR-HPV genotype and 78 (21.7%) specimens had a coinfection with two or more HR-HPV genotypes: 61 specimens with 2 genotypes, 12 specimens with 3 genotypes, and 5 specimens with 4 genotypes. Fifteen out of 378 specimens (4%) tested negative for HPV DNA with the following histopathology results: 8 HSIL, 3 squamous cell carcinoma, and 4 adenocarcinoma.

### High-risk human papillomavirus genotype distribution

HPV-16 was the most common genotype in patients with a negative histopathological finding (21/49 or 42.9% specimens); in patients with preinvasive lesions (127/219 or 57.9% specimens); and in patients with squamous cell carcinoma and adenocarcinoma (46/69 or 66.7% and 18/36 or 50%, respectively). The results for other HPV types are shown in Table 1.

There were only 5 patients with LSIL, 3 out of whom had single high-risk genotype infection and 2 had multiple infections. HPV-51 was the most common genotype (2 monoinfections and 1 multiple infection).

**Figure 1:**
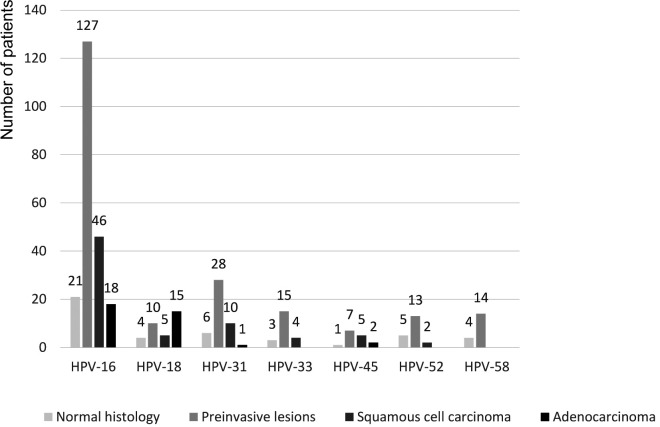
Distribution of the most common human papillomavirus (HPV) genotypes (including single and multiple infections) in specimens from women with normal histopathology, preinvasive lesions, squamous cell carcinoma, and adenocarcinoma.

Figure 1 shows the distribution of the most common HR-HPV types (including those from multiple infections) in specimens with negative histopathological findings, preinvasive lesions, squamous carcinomas, and adenocarcinomas.

HPV-16 was the most frequently detected genotype in the tested specimens regardless of the histopathological diagnosis. Overall, HPV-16 was detected in 212 of 358 specimens (59.2%); 166 monoinfections and 46 multiple infections. The prevalence of other high-risk HPV genotypes in the specimens was as follows: HPV-31 in 45 (12.6%), HPV-18 in 24 (6.7%), HPV-33 in 22 (6.1%), HPV-52 in 20 (5.6%), and HPV-58 in 18 (5.0%).

There was no significant difference in the distribution of single and multiple infections in specimens according to different histopathological diagnoses (Pearson χ^2^ test, *P* = 0.899).

### Demographic and clinical findings

The median age was as follows: preinvasive lesions group 35 years, squamous carcinoma group 46 years, adenocarcinoma group 40 years and negative histopathological findings group 35 years (Table 2). The mean parity in the preinvasive lesions group was 1.31 (SD 1.179) children, in invasive lesions group it was 1.89 children (SD 1.146), and in patients with negative histopathological findings it was 1.14 children (SD 1.099). Invasive lesions group, negative histopathological findings group, and preinvasive lesions group significantly differed in the mean age (F = 35,57, *P* ≤ 0.001), number of deliveries (F = 10.60, *P* ≤ 0.001), and smoking (F = 1250, *P* ≤ 0.001), but did not significantly differ in the number of abortions (F = 1.62, *P* = 0.19) and the age of menarche (F = 2.39, *P* = 0.09).

**Table 2:** Distribution of single and multiple human papillomavirus (HPV) infections according to patients’ age and histopathological diagnosis*

Histopathological analysis	HPV status				Age			
	negative (n)	single HPV HR (n)	multiple HPV infections (n)	LR HPV (n)	median	min	max	standard deviation
Negative	0	35	12	2	35	21	69	12.41
Low-grade squamous intraepithelial lesions	0	3	2	0	34	26	50	9.78
High-grade squamous intraepithelial lesions	8	167	43	1	35	19	70	9.02
Squamous cell carcinoma	3	52	14	0	46	27	83	11.97
Adenocarcinoma	4	25	7	0	40	21	69	11.86

*Abbreviations: HR – high-risk; LR – low-risk.

The patients were mostly from Zagreb (165 patients) and Zagreb County (72 patients, a total of 62.7%), but also from other parts of Croatia: Central Croatia 50 (13.2%), Slavonia 44 (11.6%), Dalmatia 34 (9%), and Istria, Primorje-Gorski Kotar County 13 (3.5%).

### Human papillomavirus-16 genomic variants in women with high-grade squamous intraepithelial lesions

Due to financial constraints, HPV-16 genomic variants of LCR, E6, and E7 regions were analyzed in 130 randomly selected specimens out of 212 HPV-16-positive specimens.

In total, 35 LCR variants were detected in CIN-3 specimens and one LCR variant was detected in the CIN-2 specimen (Supplementary Figure 1(Supplementary Figure 1)). Five sequences from CIN-3 specimens were identical to the reference LCR sequence. The analysis of the entire LCR sequence (823 bp, nucleotide [nt] 7155 to 82) showed one deletion and 43 nt substitutions in CIN-3 specimens and 2 nt substitutions in the CIN-2 specimen.

The most distant genetic variant from CIN-3 specimens differed from the reference HPV-16 by 12 nt, whereas the greatest variability of the LCR region in CIN-3 specimens was 1.4%. The most distant genetic variant in the CIN-2 specimen differed from the reference HPV-16 by 2 nt, and the LCR genomic region variability in the CIN-2 specimen was 0.2%. The most common variant detected in CIN-3 specimens was 16-LCR-2, with two mutations (G7193T and G7521A) found in 25/74 (33.8%) isolates. The genomic variant found in the CIN-2 specimen also had 2 mutations (T7230G and A7316G).

Eleven E6 variants were found in CIN-3 specimens (Supplementary Figure 2(Supplementary Figure 2)). The E6 variant in the CIN-2 specimen was identical to the reference sequence. Twenty-five (34.2%) sequences in CIN-3 specimens were identical to the reference E6 sequence. The analysis of the complete E6 sequence (477 bp, nt 83 to 559) showed 18 nt substitutions in CIN-3 specimens. The greatest variability of the E6 region in CIN-3 specimens was 1.67% (8 nt). HPV-16 genomic variant carrying T350G mutation was found in 45/74 (60.8%) CIN-3 specimens.

Four E7 variants were found in CIN-3 specimens (Supplementary Figure 2(Supplementary Figure 2)). The E7 variant in the CIN-2 specimen was identical to the reference sequence. Sixty-six (90.4%) sequences from CIN-3 specimens were identical to the reference E7 sequence. The analysis of the complete E7 sequence (297 bp, nt 562 to 858) showed 5 nt substitutions in CIN-3 specimens. The maximum variability of E7 genomic sequence in CIN-3 specimens was 0.6% (2 nt).

When analyzing together the LCR, E6 and E7 sequences for each individual isolate, 40 different genomic variants were found in 74 specimens from women with CIN 3 (Table 3). The sequence from the CIN-2 specimen had a variant in the LCR area, while E6 and E7 were identical to the reference sequence. Overall, 41 genomic variants were found among 74 sequences from CIN-3 and CIN-2 specimens: 73 sequences (98.6%) belonged to the European branch, while one (1.4%) variant belonged to the African branch.

**Table 3:** Genomic variants of human papillomavirus virus-16 (HPV-16) in women with cervical intraepithelial neoplasia (CIN) 3, squamous cell carcinoma, and adenocarcinoma; R variants that are identical to the reference sequence of HPV-16*

CIN 3						Squamous cell carcinoma						Adenocarcinoma					
Genomic variant (GV)	LCR variant	E6 variant	E7 variant	N	Branch	Genomic variant	LCR variant	E6 variant	E7 variant	N	Branch	Genomic variant	LCR variant	E6 variant	E7 variant	N	Branch
GV 1	LCR-R	E6-R	E7-R	5	E	GV 1	LCR-R	E6-R	E7-R	1	E	GV 1	LCR-1	E6-R	E7-1	1	E
GV 2	LCR-1	E6-1	E7-R	1	E	GV 2	LCR-1	E6-1	E7-R	2	E	GV 2	LCR-2	E6-1	E7-R	1	E
GV 3	LCR-2	E6-2	E7-R	20	E	GV 3	LCR-1	E6-2	E7-R	1	E	GV 3	LCR-3	E6-2	E7-R	1	E
GV 4	LCR-2	E6-R	E7-R	1	E	GV 4	LCR-2	E6-R	E7-R	1	E	GV 4	LCR-4	E6-3	E7-R	1	E
GV 5	LCR-2	E6-5	E7-R	1	E	GV 5	LCR-3	E6-R	E7-R	1	E	GV 5	LCR-5	E6-4	E7-2	1	AA
GV 6	LCR-2	E6-6	E7-R	1	E	GV 6	LCR-4	E6-R	E7-R	1	E	GV 6	LCR-6	E6-5	E7-R	1	E
GV 7	LCR-2	E6-7	E7-R	1	E	GV 7	LCR-5	E6-R	E7-R	1	E	GV 7	LCR-7	E6-5	E7-R	1	E
GV 8	LCR-2	E6-9	E7-R	1	E	GV 8	LCR-6	E6-R	E7-R	1	E	GV 8	LCR-8	E6-5	E7-R	1	E
GV 9	LCR-3	E6-R	E7-R	5	E	GV 9	LCR-7	E6-3	E7-R	1	E	GV 9	LCR-9	E6-R	E7-1	1	E
GV 10	LCR-4	E6-2	E7-R	1	E	GV 10	LCR-7	E6-4	E7-R	1	E	GV 10	LCR-10	E6-R	E7-R	1	E
GV 11	LCR-5	E6-2	E7-R	1	E	GV 11	LCR-7	E6-5	E7-R	1	E	GV 11	LCR-11	E6-R	E7-R	1	E
GV 12	LCR-6	E6-2	E7-R	1	E	GV 12	LCR-8	E6-6	E7-1	1	NA						
GV 13	LCR-7	E6-2	E7-R	1	E	GV 13	LCR-9	E6-R	E7-R	1	E						
GV 14	LCR-8	E6-3	E7-1	1	E	GV 14	LCR-10	E6-R	E7-R	1	E						
GV 15	LCR-9	E6-2	E7-R	1	E	GV 15	LCR-11	E6-R	E7-R	1	E						
GV 16	LCR-10	E6-4	E7-2	1	E	GV 16	LCR-12	E6-7	E7-R	2	E						
GV 17	LCR-11	E6-R	E7-R	1	E	GV 17	LCR-13	E6-R	E7-R	1	E						
GV 18	LCR-12	E6-R	E7-R	1	E	GV 18	LCR-12	E6-8	E7-R	1	E						
GV 19	LCR-13	E6-1	E7-R	1	E	GV 19	LCR-14	E6-R	E7-R	1	E						
GV 20	LCR-14	E6-R	E7-R	1	E	GV 20	LCR-15	E6-R	E7-R	1	E						
GV 21	LCR-15	E6-R	E7-1	3	E	GV 21	LCR-16	E6-R	E7-R	1	E						
GV 22	LCR-16	E6-R	E7-R	1	E	GV 22	LCR-17	E6-9	E7-R	4	E						
GV 23	LCR-17	E6-2	E7-R	1	E	GV 23	LCR-18	E6-10	E7-R	1	E						
GV 24	LCR-18	E6-R	E7-R	1	E	GV 24	LCR-19	E6-9	E7-R	1	E						
GV 25	LCR-19	E6-2	E7-R	3	E	GV 25	LCR-20	E6-R	E7-R	1	E						
GV 26	LCR-20	E6-2	E7-R	1	E	GV 26	LCR-21	E6-R	E7-R	1	E						
GV 27	LCR-21	E6-2	E7-R	1	E	GV 27	LCR-22	E6-R	E7-R	1	E						
GV 28	LCR-22	E6-R	E7-1	1	E	GV 28	LCR-23	E6-R	E7-2	1	E						
GV 29	LCR-23	E6-2	E7-R	1	E	GV 29	LCR-24	E6-R	E7-2	1	E						
GV 30	LCR-24	E6-2	E7-R	1	E	GV 30	LCR-25	E6-R	E7-R	1	E						
GV 31	LCR-25	E6-R	E7-R	1	E												
GV 32	LCR-26	E6-8	E7-3	1	Af												
GV 33	LCR-27	E6-R	E7-R	2	E												
GV 34	LCR-28	E6-2	E7-R	1	E												
GV 35	LCR-29	E6-2	E7-R	2	E												
GV 36	LCR-30	E6-R	E7-R	1	E												
GV 37	LCR-31	E6-2	E7-R	1	E												
GV 38	LCR-32	E6-10	E7-R	1	E												
GV 39	LCR-33	E6-R	E7-R	1	E												
GV 40	LCR-32	E6-1	E7-R	1	E												

*Abbreviations: E – European branch; Af – African branch; NA – North American branch; AA – Asian-American branch.

### Human papillomavirus-16 genomic variants in women with squamous cell carcinoma and adenocarcinoma

Twenty-six LCR variants were found in squamous cell carcinoma specimens and 11 LCR variants in adenocarcinoma specimens (Supplementary Figure 3(Supplementary Figure 3)). One isolate in squamous cell carcinoma specimens was identical to the reference LCR sequence. The analysis of the entire LCR region (823 bp, nt 7155 to 82) showed 34 nt substitutions in squamous cell carcinoma specimens and 27 nt substitutions in adenocarcinoma specimens.

In squamous cell cancer, the most genetically distant variant differed from the reference HPV-16 in 15 nt, and the greatest variability of the LCR genomic region specimens was 1.8%. In adenocarcinoma specimens, the most genetically distant variant differed from the reference HPV-16 in 16 nt, and the greatest variability of the LCR genomic region was 1.9%.

The most common variant in squamous cell carcinoma specimens was 16-LCR-17, with two mutations (G7193T and G7521A) found in 4/35 (11%) isolates. In adenocarcinoma specimens, all variants were equally represented (9%), and none of the isolates was identical to the reference sequence.

Eleven E6 variants were found in squamous cell carcinoma and 6 E6 variants in adenocarcinoma specimens (Supplementary Figure 4(Supplementary Figure 4)). Nineteen (54.3%) sequences from the squamous cell carcinoma specimens and 4 (36.3%) sequences from adenocarcinoma specimens were identical to the reference sequence. The analysis of the entire E6 sequence (477 bp, nt 83 to 559) showed 10 nt substitutions in squamous cell carcinoma specimens and 8 nt substitutions in adenocarcinoma specimens. The greatest variability of the E6 region in squamous cell carcinoma specimens was 1.04% (5 nt), and in adenocarcinoma specimens it was 1.25% (6 nt). The genomic variant T350G was found in 15/35 (42.8%) isolates in squamous cell carcinoma specimens and in 6/11 (54.5%) isolates in adenocarcinoma specimens.

Three E7 variants were found in squamous cell carcinoma and 3 E7 variants in adenocarcinoma specimens (Supplementary Figure 4Supplementary Figure 4(Supplementary Figure 4)). Thirty-two (91.4%) sequences from squamous cell carcinoma specimens and 8 (72.7%) sequences from adenocarcinomas were identical to the reference E7 sequence. The analysis of the entire E7 sequence (297 bp, nt 562 to 858) showed 3 nt substitutions in squamous cell carcinoma specimens and 4 nt substitutions in adenocarcinoma specimens. The greatest variability of the E7 region in squamous cell carcinoma specimens was 0.6% (2 nt), and in adenocarcinoma specimens it was 1.0% (3 nt).

When analyzing together the LCR, E6, and E7 sequences for each individual isolate in 35 squamous cell carcinoma specimens, 30 different genomic variants were found (Table 3). When analyzing together the LCR, E6, and E7 sequences in 11 adenocarcinoma specimens, 11 different genomic variants were found (Table 2).

Overall, 44 (95%) isolates in carcinoma specimens grouped into 41 genomic variants belonged to the European branch, one isolate (2.5%) belonged to the North American branch (one specimen of squamous cell carcinoma), and one (2.5%) belonged to the Asian-American branch (one adenocarcinoma specimen).

### Human papillomavirus-16 genomic variants in patients with negative histopathological finding

In negative histopathological specimens, 6 LCR variants were found. No sequences were identical to the reference LCR sequence. The analysis of the complete LCR sequence (823 bp, nt 7155 to 82) showed 6 nt substitutions. The greatest variability of the LCR region was 0.36% (3 nt). HPV-16 genomic variant carrying G7193T and G7521A mutations was found in 5/10 (50.0%) specimens.

Two E6 variants were found. Two sequences were identical to the reference E6 sequence. The analysis of the complete E6 sequence (477 bp, nt 83 to 559) showed 1 nt substitution. The greatest variability of the E6 region was 0.21% (1 nt). HPV-16 genomic variant carrying T350G mutation was found in 8/10 (80.0%) specimens.

All E7 variants were identical to the reference sequence. When analyzing together the LCR, E6, and E7 sequences for each individual isolate, 6 different genomic variants were found in 10 specimens.

## Discussion

 In our study, the most frequent HPV genotype in women from Croatia with histologically confirmed HSIL and cervical cancer was HPV-16. HPV-16 variants dominantly belonged to the European branch regardless of the pathohistological diagnosis. Persistent infection with one or more HPV genotypes with high oncogenic potential has been identified as the most important factor causing cervical neoplasia. At least 12 HR-HPV types have been recognized as carcinogenic: HPV-16, HPV-18, HPV-31, HPV-33, HPV-35, HPV-39, HPV-45, HPV-51, HPV-52, HPV-56, HPV-58, and HPV-59. The prevalence of HR-HPV types varies geographically, with HPV-16 being the most common genotype in premalignant and malignant lesions worldwide.

Cervical cancer is the ninth cause of malignancy in women in Croatia, as at least 300 women develop cervical cancer every year. The prevalence of HR-HPV in the tissue of HSIL and cervical carcinoma has not been systematically studied yet.

Several studies assessed the prevalence of different HPV genotypes, but this was done mostly in the swab specimens, rarely involving histopathological examination. In several cases, HPV DNA was detected in a relatively small number of archival paraffin embedded cervical cancer specimens (20-27). Grce et al in 1997 (21) analyzed 379 scrape specimens from women with previously documented Pap smear cytology classified from 1-4 by in-house PCR method, including consensus primer pair MY09/MY11 and specific primer pairs for HPV6/11, 16, 18, 31, and 33. The study population included 61 specimens with CIN 2, 50 specimens with CIN 3 cytology, and only 18 with CIN 4. However, only 50% of CIN-2 and CIN-3, and 60% of CIN-4 specimens tested positive for HPV. The most commonly detected HPV genotype in HSIL and carcinoma lesions was HPV-16 (18%), followed by HPV-31 in 13% and HPV-18 in only 6% of the cases. Histopathology data were unavailable because of design and methodological limitations, so the study shows only preliminary genotype distribution. Our study, on the contrary, had histopathology evidence for 100% of participants, and HPV was detected in 95% of cases. Fifteen specimens (4%) were HPV-negative (8 HSIL, 3 squamous cell carcinoma, and 4 adenocarcinoma specimens). This is consistent with the previous studies that confirmed the existence of HPV-negative carcinomas (3,33-35), a phenomenon that could be explained by low HPV DNA content, loss of HPV within the tumor, misdiagnosed tumor of another origin, inability of the test to determine certain HPV genotypes, and the existence of cervical cancers independent of HPV.

Milutin-Gasperov et al in 2007 (25) assessed the prevalence of high-risk HPV genotypes among Croatian women by using archival cervical swab DNA specimens from women with a spectrum of various cytological diagnoses from normal cytology to HSIL (41%), with a very high proportion (25%) of patients with atypical cells of undetermined significance (ASCUS). Overall, HPV DNA was detected in 58.8% of specimens, 64% of which were further genotyped. However, HPV type could not be determined in 35.5% of specimens. In HSIL specimens, the most prevalent type was HPV-16 (23% of cases), followed by HPV-31 (12%), HPV-33 (6.1%), HPV-18 (5%), HPV-52 (2.3%), HPV-58 (1.1%), and HPV-45 (0.9%). Multiple infections were found in 10% of cases. Again, histopathology data were unavailable, and the large number of ASCUS results, which is usually below 5% of cytology findings, indicates that the cytology findings came from different laboratories with different expertise level. In addition, there were no specimens from patients with cervical cancer, so the results were of limited value.

Kaliterna et al in 2007 (22) performed the HPV DNA typing among general population of women from Split-Dalmatia County with unknown cytological diagnosis. Out of 570 tested women, 200 (35%) were HR-HPV positive, with HPV-16 as the most abundant type in 28.5% of the patients, followed by HPV-18 (17.5%), HPV-31 (7.5%), HPV-33 (5.5%), HPV-52 (4%), HPV-59 (2%), and HPV-45 (1%). A large proportion of specimens were of an unresolved genotype (34%).

The same authors in 2013 (23) assessed the HR-HPV prevalence in women from Split-Dalmatia County with reference to cytology results. Out of 1160 tested women, 406 (35%) were HR-HPV positive and 12.2% of all tested samples had an unresolved genotype. Out of those 406 positive results, 148 (36.5%) had HSIL, and a high proportion of women had ASCUS/LSIL – 148 (45.8%). HPV-16 was the most frequent type within each cytological category, with a prevalence of 30.8%. HPV prevalence within the HSIL category was as follows: HPV-16 in 34.5%, HPV-18 in 22.3%, HPV-31 in 6.8%, HPV-33 in 3.4%, HPV-52 and HPV-59 in 2% each, and HPV-45 in 1.4% specimens. There were no histopathological data.

A pilot study by Roksandic-Krizan et al (26) from 2013 conducted in northeast Croatia in 100 women with abnormal cervical cytology analyzed high-risk/intermediate-risk/low-risk HPV prevalence. The authors found multiple infections in 43% specimens, with the most prevalent HR genotypes being HPV-16 (27.6%), HPV-31 (11.8%), HPV-51, and HPV-52 (10.2% each). No data regarding HR-HPV distribution were available within each cytological category, but the authors identified a low number of HSIL cases as the main study limitation.

Marijan et al (24) determined the HR-HPV prevalence in different age groups of women from the Zagreb region. A total of 3440 samples were tested, but the information regarding cervical abnormalities was unavailable for 48% of the specimens. A total of 34% of specimens were identified as ASCUS and only 4.4% samples as HSIL. The HR-HPV prevalence was 34.6%, and it decreased with age. The study limitation was a high proportion of younger patients (44.2% aged 21-30). No data regarding distribution of different HPV genotypes were available.

Grahovac et al (20) explored the HR-HPV prevalence and HPV genotypes among 361 women attending regular gynecological visits. Seventy-two out of 361 women (19.9%) were diagnosed with HSIL, other were without abnormal lesions (56.8%) or had ASCUS/LSIL (23.3%). HR-HPV prevalence increased with the severity of cytological diagnosis, and women with HSIL had HR-HPV prevalence 80.6%. HPV-16 was predominant in all cytological entities. In women with HSIL it was present in 63.8% of patients, while other most frequent types were as follows: HPV-31 (8.6%), HPV-33 (6.9%), and HPV-18 5.2%. There were 10.3% women with indeterminate HPV type. Again, as in other mentioned studies no information regarding histopathology was available.

Our results support the results of Sabol et al (27) that HPV-16 was the most common genotype in HSIL, followed by HPV-31, HPV-58, HPV-33, and HPV-52. However, we cannot directly compare the distribution of HPV genotypes obtained in our study with that obtained by Sabol et al due to a small number of patients with histologically confirmed cancer in their study (N = 35), especially with reference to the distribution difference depending on the histological type of cancer.

The present study for the first time explored the genomic heterogeneity of HPV-16, the most common causative agent of HSIL and cervical carcinoma in Croatian patients. It is assumed that some variants carry an increased oncogenic potential. In our study, mainly European genomic variants of HPV-16 were detected both in HSIL and carcinoma specimens.

Similar to our findings, LCR was found to be the most variable segment of the HPV-16 genome in different populations (36,37). At the same time, HPV E7 was highly conserved. The T350G genomic variant of HPV-16 was found in 62.5% of HSIL specimens, in 42.8% of squamous cell carcinoma specimens, and in 63.6% of adenocarcinoma specimens.

Some previous studies (38-41) have indicated a stronger oncogenic potential of non-European genomic variants, while others (42-44) did not support this finding. A German study showed that the oncogenic potential of Asian-American or North American lineages was influenced by polymorphisms in the LCR and possibly other viral genome regions. In the European lineage, this phenomenon appears to be associated with E6 rather than LCR variations (18). In European HPV-16 isolates, polymorphisms in the LCR are more frequent than in the E6 gene (18).

A Slovenian study (45) reported non-European variants only in 5% of the cervical carcinoma specimens, which confirms the predominance of European variants in ethnically homogeneous female populations in Europe.

The present study presents crucial information about the genetic diversity of HPV-16 in southeastern Europe. HPV-16 is the most oncogenic genotype worldwide, and the genomic variants detected in cervical carcinoma specimens represent the most oncogenic variants. The main limitation of our study is a relatively small number of analyzed specimens due to restricted financial resources.

Our results support the previous findings that mostly European HPV-16 variants are discovered in small European populations that are not mixed with different ethnical groups. Nevertheless, further studies are necessary to give more information about the genomic diversity of HPV-16 in cervical carcinoma patients in this part of Europe.

Cervical cancer is the tenth cancer in terms of incidence in Croatian women (46). It is also the third most common cancer among women of fertile age. Therefore, this is a significant public health issue that negatively affects both women's reproductive health and demographic statistics. In 2014, 307 cases of cervical cancer were newly diagnosed, with the highest rate in women aged 45-59 (86.1%) (46). HSIL incidence is the highest in the age-group 30-34, which was the group with the largest share of births in Croatia in 2016 (34%) (47).

Furthermore, as the number of newly discovered patients has not declined since 2010 despite the introduction of the National Early Cervical Cancer Screening Program, there is need for a comprehensive national strategy of cervical cancer prevention to further reduce the incidence of cervical cancer and preinvasive lesions in Croatia. Currently available vaccines, especially the most recent nine-valent vaccine, cover all the most common HPV types detected in Croatian women with histologically proven lesions (HSIL or cancer), suggesting that this vaccine could be an excellent primary prevention tool in Croatia.
