# Supplementary material for: Distribution of human papillomavirus genotypes in women with high-grade cervical intraepithelial lesions and cervical carcinoma and analysis of human papillomavirus-16 genomic variants
Source: Croat Med J. 2021 Feb;62(1):68–79. doi: 10.3325/cmj.2021.62.68 (PMC7976879; doi:10.3325/cmj.2021.62.68)
Supplement: Supplementary Figure 2 [file CroatMedJ_62_s005.pdf]

		E6																									
		83	93	109	131	132	134	143	145	176	182	185	188	216	233	256	286	289	335	350	373	395	412	473	532	535	*f*
**Reference**	**16-E6-R**	a	a	t	a	g	a	c	g	g	a	t	g	t	g	c	t	a	c	t	g	g	t	a	a	a	25
**CIN 3**	16-E6-1			c																g							3
	16-E6-2																			g							37
	16-E6-3						c																				1
	16-E6-4				g																						1
	16-E6-5																			g			c				1
	16-E6-6					t							c							g							1
	16-E6-7													c						g							1
	16-E6-8	c				c		g	t								a	g	t					c			1
	16-E6-9									c										g							1
	16-E6-10																			g	a						1
CIN 2	16-E6-1																										1

		E7						
		712	732	789	795	802	822	*f*
**Reference**	**16-E7-R**	c	t	t	t	g	a	65
**CIN 3**	16-E7-1						g	5
	16-E7-2	a				a		1
	16-E7-3			c	g			1
**CIN2**	16-E7-R							1
