# Supplementary material for: Distribution of human papillomavirus genotypes in women with high-grade cervical intraepithelial lesions and cervical carcinoma and analysis of human papillomavirus-16 genomic variants
Source: Croat Med J. 2021 Feb;62(1):68–79. doi: 10.3325/cmj.2021.62.68 (PMC7976879; doi:10.3325/cmj.2021.62.68)
Supplement: Supplementary Figure 4 [file CroatMedJ_62_s007.pdf]

		E6																									
**HPV16**		83	93	109	131	132	134	143	145	176	182	185	188	216	233	256	286	289	335	350	373	395	412	473	532	535	f
**Reference**	**16-E6-R**	a	a	t	a	g	a	c	g	g	a	t	g	t	g	c	t	a	c	t	g	g	t	a	a	a	19
**SCC**	16-E6-1																			g							2
	16-E6-2										c									g							1
	16-E6-3																			g						g	1
	16-E6-4																			g							1
	16-E6-5															t				g							1
	16-E6-6								t								a	g	t	g							1
	16-E6-7																			g							2
	16-E6-8		g																								1
	16-E6-9																			g							5
	16-E6-10														a					g							1

		E6																									
**HPV16**		83	93	109	131	132	134	143	145	176	182	185	188	216	233	256	286	289	335	350	373	395	412	473	532	535	f
**Reference**	**16-E6-R**	a	a	t	a	g	a	c	g	g	a	t	g	t	g	c	t	a	c	t	g	g	t	a	a	a	4
**AC**	16-E6-1																			g		t					1
	16-E6-2																			g							1
	16-E6-3											g															1
	16-E6-4								t								a	g	t	g					g		1
	16-E6-5																			g							3

		E7						
**HPV16**		712	732	789	795	802	822	f
**Reference**	**16-E7-R**	c	t	t	t	g	a	32
**SCC**	16-E7-1			c	g			1
	16-E7-2						g	2
**HPV16**		712	732	789	795	802	822	f
**Reference**	**16-E7-R**	c	t	t	t	g	a	8
**AC**	16-E7-1						g	2
	16-E7-2		c	c	g			1
