# Supplementary material for: Distribution of human papillomavirus genotypes in women with high-grade cervical intraepithelial lesions and cervical carcinoma and analysis of human papillomavirus-16 genomic variants
Source: Croat Med J. 2021 Feb;62(1):68–79. doi: 10.3325/cmj.2021.62.68 (PMC7976879; doi:10.3325/cmj.2021.62.68)
Supplement: Supplementary Figure 1 [file CroatMedJ_62_s004.pdf]

| | | LCR | | | | | | | | | | | | | | | | | | | | | | | | | | | | | | | | | | | | | | | | | | | | | | | | | | | | | | | | | | | | | | | |
|---|---|---|---|---|---|---|---|---|---|---|---|---|---|---|---|---|---|---|---|---|---|---|---|---|---|---|---|---|---|---|---|---|---|---|---|---|---|---|---|---|---|---|---|---|---|---|---|---|---|---|---|---|---|---|---|---|---|---|---|---|---|---|---|---|---|---|---|
| | | 7172 | 7174 | 7185 | 7193 | 7230 | 7232 | 7233 | 7287 | 7293 | 7302 | 7310 | 7316 | 7337 | 7339 | 7350 | 7359 | 7360 | 7374 | 7377 | 7388 | 7394 | 7395 | 7429 | 7434 | 7441 | 7450 | 7451 | 7469 | 7485 | 7489 | 7496 | 7501 | 7507 | 7521 | 7549 | 7552 | 7576 | 7636 | 7669 | 7689 | 7714 | 7729 | 7730 | 7743 | 7752 | 7756 | 7764 | 7768 | 7784 | 7786 | 7792 | 7799 | 7801 | 7834 | 7868 | 7876 | 7886 | 7904 | 12 | 13 | 16 | 24 | 31 | 43 | 80 | f |
| Reference | **16-LCR-R** | t | a | t | g | t | a | a | a | t | t | c | a | c | a | t | g | g | a | a | c | c | c | g | c | t | t | c | t | a | g | t | a | a | g | c | g | a | a | c | c | t | a | a | t | a | t | c | g | c | c | c | g | a | g | g | c | c | t | t | c | g | c | c | c | t | 5 |
| CIN 3 | 16-LCR-1 | | | | t | | | | | | | | | | | | | | | | | | | | | | c | | | | | | | | a | | | | | | | | | | | | | | | | | | | | | | | | | | | | g | | | | 1 |
| | 16-LCR-2 | | | | t | | | | | | | | | | | | | | | | | | | | | | | | | | | | | | a | | | | | | | | | | | | | | | | | | | | | | | | | | | | | | | | 25 |
| | 16-LCR-3 | | | | | | | | | | | | c | | | | | | | | | | | | | | | | | | | | | | | | | | | | | | | | | | | | | | | | | | | | | | | | | | | | | | 5 |
| | 16-LCR-4 | | | | t | | | | | | | | | | | | | | | | | | | | | | | | | | | | | | a | | | | c | | | | | | | | | | | | | | | | | | | | | | | | | | | | 1 |
| | 16-LCR-5 | | | | | | | | | | | | | | | | | | | | | | | | | | | | | | | | | | a | | a | | | | | | | | | | | | | | | | | | | | | | | | | | t | | | | 1 |
| | 16-LCR-6 | | | | t | | | | | | | | | | | | | | | | | | | | | | c | | | | | | | | a | | | | | | | | | | | | | | | | | | c | | | | | | | | | | | | | | 1 |
| | 16-LCR-7 | | | | t | | | | | | | | | | | | | | | | | | | | | | | | | | | | | | a | | | | | | | | | | | | | | | | | | | | | | | | | | | | g | | | | 1 |
| | 16-LCR-8 | | | | t | | | | | | | | | | | | | | | | | | | | | | | | | | | c | | | a | | | | | | | | | | | | | | | | | | | | | | | | | | | | | | | c | 1 |
| | 16-LCR-9 | | | | t | | | | | | | | | | | | | | | | | | | t | | | | | | | | | | | a | | | | | | | | | | | | | | | | | | | | | | | | | | | | | | | | 1 |
| | 16-LCR-10 | | | | t | | | | | | c | g | | | | | | | | | | | | | | | | | | | | | | | a | | | | | | | | | | | | | | | | | | | | | | | | c | | | | | | | | 1 |
| | 16-LCR-11 | | | | t | | | | | | | | | | | | | | | | | | | | | | | | | | | | | | a | | | | | | | | | | | | | | | | | | | | | a | | | | | | | | | | | 1 |
| | 16-LCR-12 | | | | | | | | | | | | c | | | | | | | | | | | | | | | | | | | | | | | | | | | | | | | | | | | | | | | | | | | | | | | | t | | | | | | 1 |
| | 16-LCR-13 | | | | t | | | | | | | | | | | | | | | | | | | | | | c | | | | | | | | a | | | | | | | | | | | | | | | | | | | | | | | | | | | | g | | | | 1 |
| | 16-LCR-14 | | | | | | | | | | | | c | | | | | | | | | | | | | | | | | | | | | | | | | | | | | | | | | | | | | | | g | | | | | | | | | | | | | | | 1 |
| | 16-LCR-15 | | | | t | | | | | | | | | | | | | | | | | | | | | | | | | | | c | | | a | | | | | | | | | | | | | | | | | | | | | | | | | | | | | | | | 3 |
| | 16-LCR-16 | | | | | | | | | | | | c | | | | | | | | | | | | | | | | | | | | | | | t | | | | | | | | | | | | | | | | | | | | | | | | | | | | | | | | 1 |
| | 16-LCR-17 | | | | t | | | | | | | | | | | | | | | | | t | | | | | | | | | | | | | a | | | | | | | | | | | | | | | | | | | | | | | | | | | | | | | | 1 |
| | 16-LCR-18 | | | | t | | | | | | | | | | | | | | | | | | | | | | | | | | | | | c | a | | | | | | | | | | | | | | | | | | | | | | | | | | | | | | | | 1 |
| | 16-LCR-19 | | | | t | | | | | | | | | | | | | | | | | | | | | | c | | | | | | | | a | | | | | | | | | | | | | | | | | | | | | | | | | | | | | | | | 3 |
| | 16-LCR-20 | | | | t | | | | | | | | | | | | | | | | | | | | | | | t | | | | | | | a | | | | | | | | | | | | | | | | | | | | | | | | | | | | | | | | 1 |
| | 16-LCR-21 | | | | . | | | | | | | | | | | | | | | | | | | | | | | t | | | | | | | a | | | | | | | | | | | | | | | | | | | | | | | | | | | | | | | | 1 |
| | 16-LCR-22 | | | | t | | | | | | | | | | | | | | | | | | | | | | | | | | | c | | | a | | | | | | | | | c | | | | | | | | | | | | | | | | | t | | | | | | 1 |
| | 16-LCR-23 | | | | t | | | | | | | | | | | | | | g | | | | | | | | | | | | | | | | a | | | | | | | | | | | | | | | | | | | | | | | | | | | | | | | | 1 |
| | 16-LCR-24 | | | | t | | | | | | | | | | | | | | | | t | | | | | | | | | | | | | | a | | | | | | | | | | | | | | | | | | | | | | | | | | | | | | | | 1 |
| | 16-LCR-25 | | | | t | | | | | | | | | | | | | | | | | | | | | | | | | | | | | | a | | | | | | | | | | | | | | | | | a | | | | | | | | | | | | | | | 1 |
| | 16-LCR-26 | | | | t | | g | | | g | | | | | | | | | | | | | | | | | | | | | a | | | | a | | | | | | a | a | | | | | | t | | | t | | | | t | | a | | | | | | | t | | | 1 |
| | 16-LCR-27 | | | | t | | | | | | | g | | | | | | | | | | | | | | | | | | | | | | | a | | | | | | | | | | | | | | | | | | | | | | | | | | | | | | | | 2 |
| | 16-LCR-28 | | c | | t | | | | | | | | | | | | | | | | | | | | | | c | | | | | | | | a | | | | | | | | | | | | | | | | | | | | | | | | | | | | | | | | 1 |
| | 16-LCR-29 | | | | t | | | | | | | | | | | | | | | | | | | | | | c | | | | | | | | a | | | | | | | | | | | | | | | | | | | | | a | | | | | | | | | | | 2 |
| | 16-LCR-30 | | | | | | | | | | | | c | | | g | | | | | | | | | | | | | | | | | | | | | | | | | | | | | | | | | | | | | | | | | | | | | | | | | | | 1 |
| | 16-LCR-31 | | | | t | | | | | | | | | | | | | | | | | | | | | | | | | | | | | | a | | | | | | | | | | | | | | | | | | | | | | | | | | | | | | t | | 1 |
| | 16-LCR-32 | | | | t | | | | | | | | | | | | | | | | | | | | | | | | | | | | | | a | | | | | | | | | | | | | | | | | | | | | | | | | | g | t | | | | | 1 |
| | 16-LCR-33 | | | | t | | | | | | | | | | | | | | | | | | | | | | | | | | | | | c | a | | | | | | | | | | | | | | | | | | | | | | | | a | | | | | | | | 1 |
| | 16-LCR-34 | | | | t | | | | | | | | | | | | | | | | | | | | | | | | | | | | c | | a | | | | | | | | | | | | | | | | | | | | | | | | | | | | | | | | 1 |
| **CIN2** | 16-LCR-1 | | | | | g | | | | | | | g | | | | | | | | | | | | | | | | | | | | | | | | | | | | | | | | | | | | | | | | | | | | | | | | | | | | | | 1 |
